# Effectiveness of Patients’ Education and Telenursing Follow-Ups on Self-Care Practices of Patients With Diabetes Mellitus: Cross-Sectional and Quasi-Experimental Study

**DOI:** 10.2196/67339

**Published:** 2025-03-21

**Authors:** Mohammed Alsahli, Alaa Abd-alrazaq, Dalia M Fathy, Sahar A Abdelmohsen, Olfat Abdulgafoor Gushgari, Heba K Ghazy, Amal Yousef Abdelwahed

**Affiliations:** 1 Health Informatics Department, College of Health Sciences, Saudi Electronic University, Riyadh, Saudi Arabia; 2 AI Center for Precision Health, Weill Cornell Medicine-Qatar, Al Luqta St, Ar-Rayyan, PO Box 5825, Doha, Qatar, +974 55708549; 3 Community Health Nursing Department, Faculty of Nursing, Kafrelsheikh University, Kafrelsheikh, Egypt; 4 Nursing Department, North Private College of Nursing, Arar, Saudi Arabia; 5 Department of Nursing Science, College of Applied Medical Sciences, Prince Sattam Bin Abdulaziz University, Wadi Aldawaser, Saudi Arabia; 6 Department of Medical-Surgical Nursing, Faculty of Nursing, Assiut University, Assiut, Egypt; 7 Public Health Department, College of Health Sciences, Saudi Electronic University, Jeddah, Saudi Arabia; 8 Public Health Department, College of Health Sciences, Saudi Electronic University, Dammam, Saudi Arabia; 9 Community Health Nursing Department, Faculty of Nursing, Damanhour University, Damanhour, Egypt

**Keywords:** diabetes mellitus, education, knowledge, self-care, telenursing

## Abstract

**Background:**

Information and communications technology can be used in telenursing to facilitate remote service delivery, thereby helping mitigate the general global nursing shortage as well as particular applications (eg, in geographically remote communities). Telenursing can thus bring services closer to end users, offering patient convenience and reduced hospitalization and health system costs, enabling more effective resource allocation.

**Objective:**

This study aims to examine the impact of patients’ education and telenursing follow-ups on self-care indicators among patients with type I and type II diabetes mellitus (DM).

**Methods:**

In phase I, a cross-sectional descriptive analysis was conducted to evaluate the self-care practices of 400 patients with DM at Kafr El Sheikh University Hospital in Egypt. In phase II, a pretest-posttest experiment was applied with a selected group of 100 patients purposively recruited from phase I due to their low self-care practice knowledge to ascertain the impacts of a 4-week intervention delivered via telenursing. They were reminded via telephone follow-up communication of the importance of adhering to recommendations on physical activity, nutritional intake, and the management of blood sugar (ie, insulin). Data collection was undertaken using a structured quantitative questionnaire, encompassing sociodemographic characteristics, medical symptoms and history, and knowledge of DM. Paired *t* test analysis was applied to study pre- and postintervention self-care behaviors.

**Results:**

Participants had a mean age of 49.7 (SD 11.5) years. More than one-third received their DM diagnosis over a decade previously (135/400, 33.8%) and were obese (147/400, 36.8%). Almost half (176/400, 44%) received insulin, and the majority had cardiac disease (231/400, 57.7%) and the DM symptom of elevated blood sugar levels while fasting (365/400, 91.3%). A relatively high score of DM knowledge was reported (255/400, 63.7%). Males exhibited significantly lower knowledge levels (102/200, 51%) compared to females (153/200, 76.5%; *P*<.001). The intervention was effective in improving knowledge of DM (*t*_99_=30.7, two-tailed; *P*<.001), self-care practices (*t*_99_=53.7, two-tailed; *P*<.001), and self-care skills (*t*_99_= 47, two-tailed; *P*<.001) among patients with DM.

**Conclusions:**

The emergent evidence suggests that patients’ education and telenursing follow-ups have the potential to improve self-care behavior in patients with DM. The delivery of frequent nursing reinforcement via telenursing enables improved self-management while contemporaneously reducing the need for patients to visit clinical settings (ie, improving patient condition and reducing net health system costs). The outcomes of this research underscore the need to integrate telenursing within conventional care for DM, and more research is needed to longitudinally assay its efficacy and sustainability over the long term and in different clinical and geographical contexts.

## Introduction

Diabetes mellitus (DM) is a chronic metabolic disorder characterized by elevated blood glucose levels resulting from defects in insulin secretion, insulin action, or both. Insulin is a hormone produced by the pancreas that regulates blood sugar levels and facilitates the uptake of glucose into cells for energy. There are 3 main types of DM (type I DM, type II DM, and gestational DM), each with etiological and pathological characteristics. Type I DM is a condition of the autoimmune system, arising from the lack of functioning beta cells generating insulin. Type II DM is more common and is generally attributable to lifestyle attributes and nutritional factors (eg, sedentary behavior and high sugar consumption), albeit genetic predispositions are also instrumental. Gestational DM occurs during pregnancy and typically resolves after delivery, although it increases the mother’s risk of developing type II diabetes later in life.

DM poses a significant threat to the safety of hundreds of millions of people worldwide, with disconcertingly escalating prevalence. It is estimated that 643 million people will be diagnosed by 2030, rising to 783 million by 2045, up from 537 in 2021 [1]. This estimated increase can be associated with global population growth and the rising prevalence of diabetes due to unhealthy lifestyle-related factors and aging populations. The prevalence is significantly higher in certain regions, including the Middle East, where more than 70 million people are currently affected by DM. According to the International Diabetes Federation, Egypt ranks ninth globally for DM prevalence. In early 2020, there were approximately 8.85 million people with DM in the country, representing a prevalence rate of 15.2% [2].

DM entails direct costs in itself, and it also entails secondary costs related to interlinked conditions (which may themselves be causative or reciprocally exacerbated by DM). DM is often associated with complications such as vision impairment and blindness, cardiovascular diseases, and kidney failure and may require foot amputation [3]. In order to mitigate the more serious impacts of the condition and enable patients to have a better quality of life, DM must be managed with a strong autonomous role of patients themselves, including consistent adherence to practices recommended for self-care, such as frequent monitoring of their blood glucose levels, appropriate nutritional intake, recommended levels of physical activity, and medication compliance [3].

While patients tend to be aware of the imperatives associated with such positive behaviors, they commonly struggle to implement them in their daily lives, especially as metabolic disorders and DM itself commonly arise from a knowing lack of compliance with positive behaviors (ie, the general public typically knows that eating large amounts of processed sugar and having a sedentary lifestyle will predispose them to DM, yet they continue to indulge in such behaviors, leading to or exacerbating diabetes) [3]. In low-income countries, research has consistently shown that a large proportion of patients with DM typically adhere to negative self-care, essentially manifesting poor control of their glycemic index and a commensurately elevated propensity toward serious resultant issues [4,5].

The accelerating development and adoption of many useful technological solutions in health care services over the last 2 decades have led to greatly expanded opportunities for the more effective management of chronic illness, including DM. Telenursing, which is defined as the use of technological channels (eg, telephone or video calls) to provide nursing services to individuals in remote locations, has offered ways in which to reduce the distance between health care services and patients, as well as reducing the need for some patients to attend traditional care venues (thereby reducing pressure on limited resources) [6]. Its obvious advantages include increasing health providers’ interaction with service users, including for symptoms monitoring and educating service users without expensive and burdensome face-to-face clinical appointments.

Telenursing fundamentally increases the ability of health care professionals to deliver services remotely, which has obvious implications for more frequent monitoring of patient symptoms and escalating interventions where appropriate, with personalized assistance for service users in the comfort of their homes and everyday lives [7]. As DM management is particularly sensitive to general lifestyle factors, the telenursing paradigm can be particularly useful to extend the reach of health care providers to give patients with DM additional support and encouragement in their daily lives, especially with engagement for reminders and follow-up on particular issues [7].

It should be noted that telenursing benefits encompass important clinical outcomes in addition to practice expedience in communication; the more frequent and direct communication engendered by telenursing formats enables increased patient adherence to medication, self-care, and other outcomes, which intrinsically comprises improved quality of care and contributes to optimized patient prognosis [8]. A systematic review found that telenursed patients displayed statistically significant enhancement in their glycemic control, with 0.5% reduced HbA_1c_ (glycated hemoglobin A_1c_) levels over half a year, alongside decreased BMI in some studies that effectively leveraged “combined” interventions [9].

Additionally, telenursing mitigates the burden placed on health services by obviating in-person (face-to-face) attendance at traditional care delivery venues, which is especially valuable in resource-constrained contexts, such as low-income countries or remote geographical regions [10]. In areas suffering from a dearth of conventional health care resources, telenursing offers essential care delivery channels for patients with DM, preventing the escalation of patients’ conditions and reducing net health care costs (eg, timely telenursing interventions can reduce the need for hospital admission) [11].

Among the particular services that can be enhanced by telenursing, limited research has explored its potential to play a role in improving DM patients’ capacity to undertake self-care practices. It appears to offer notable advantages, but differing results have been found in practice, with some studies reporting tangible positive outcomes, and others identifying substantive barriers in terms of technological issues and the stakeholder engagement, which can hamper the long-term sustainability of telenursing services [8]. A recent narrative review of 18 randomized controlled trials (RCTs) and 5 quasi-experimental studies worldwide concerning telenursing for DM care reported that a telenursing intervention of weekly telenursing contact over 3 months achieved no significant influence on BMI or weight loss, while a 6-month telenursing program attained no significant differences in either BMI or HbA_1c_ [8]. A systematic review of adherence to medication regimens among patients with DM found that there was no study that had reported consistent improvement due to telenursing [12]. Such negative findings are contrary to expectations, given the potential promise of telenursing; thus, further studies are needed to ascertain telenursing impacts on self-care practices among patients with DM in numerous different and varied health care settings.

This study seeks to fill this research gap by ascertaining the impacts of patients’ education and telenursing follow-ups on self-care practices among patients with DM at an Egyptian tertiary hospital. Using a single-group pretest-posttest design and cross-sectional analytical approaches, this research sought to evidence telenursing’s scope to enhance self-care practices, thereby improving the quality of care and outcomes for patients with DM. The outcomes can guide practice in clinical contexts and advance emerging studies on digital health solutions for the management of chronic diseases, especially DM. The insights gained from this research are particularly important in considering the intervention impacts to improve self-care practices among patients with DM, especially for contexts where conventional care is limited or hard to access.

## Methods

### Study Design

This 2-phase study encompassed a cross-sectional assessment of self-care (phase I) and a single-group quasi-experimental pretest-posttest design to assess the impacts of telenursing education on patients’ knowledge, skills, and self-care (phase II).

### Study Setting

The research setting was the outpatient clinic for diabetes care at Kafr El Sheikh University Hospital. This is the main diabetes care hub for the whole governorate. The sessions for patient education were delivered in specially allocated locations within the clinic, and the phase II follow-up interventions were delivered remotely using WhatsApp or SMS text messages.

### Sampling

#### Inclusion Criteria

To be eligible for the study, participants had to (1) be adult patients with a diagnosis of DM for at least 1 year, (2) be aged between 18 and 65 years, (3) have access to and the ability to use a smartphone, (4) have HbA_1c_ level greater than 7, and (5) express interest in and willingness to participate in the study’s interventions. Patients were excluded if they had psychological illnesses, speech or hearing impairments, or failed to respond to mobile phone contact for 2 weeks.

#### Sample Size and Sampling Technique

For phase I, clinical records for outpatients during 2020 were analyzed. The outpatient clinic records for the year 2020 were reviewed to determine the patient population. Using the Roasoft calculation program with a 50% response rate, a 95% CI, and a 5% margin of error, the required sample size was calculated to be 384. In order to attain more robust data, we purposively selected 400 eligible patients who met the inclusion criteria (above), comprising 200 males and 200 females.

The preliminary analysis of the data collected in phase I showed that 255 (63.7%) of 400 patients were categorized as having poor knowledge and poor self-care practice. Based on the inclusion criteria, a purposive sample of 100 patients was selected for phase II, focusing specifically on those with the lowest scores in both knowledge and self-care practices, as they were identified as the patients most in need of educational intervention.

### Data Collection Tools

#### Sociodemographic and Medical Data Questionnaire

This tool gathered data on sociodemographic features such as age, educational level, and marriage status, and clinical attributes such as time since diabetes diagnosis, presence and type of comorbidities, fasting blood glucose levels, and BMI.

#### Knowledge Assessment Questionnaire

Participants’ knowledge about DM (hereinafter “knowledge”) was gathered using 23 open-ended questions divided into 8 categories: basic knowledge about diabetes and its complications (10 questions), treatment regimens (3 questions), physical exercise (2 questions), the importance of follow-up visits (2 questions), dietary patterns (2 questions), foot care (2 questions), bad habits that worsen the disease (1 question), and sources of knowledge (1 question). Responses were scored using a system where correct and complete answers received 5 marks, correct but incomplete answers received 4 marks, incomplete answers received 3 marks, incorrect answers received 2 marks, and answers of “don’t know” received 1 mark. Each subsection score was averaged, with total knowledge scores ranging from 23 to 115 marks. Scores were then classified into poor knowledge (less than 60%, ≤69 marks) and fair knowledge (60% or more, ≥70 marks).

#### Self-Care Practices Questionnaire

This questionnaire focused on self-care practices among patients with DM and covered 43 different practices, which were categorized into 6 areas: nutritional practices and adherence to the DM dietary regimen (12 practices), practices related to medication regimen (5 practices), practices related to glucose monitoring (6 practices), practices related to physical activity (8 practices), practices to avoid complications (6 practices), and practices related to foot care (6 practices). The self-care practices were assessed using a 3-point Likert scale, where responses were rated as “always” (2 marks), “sometimes” (1 mark), and “rarely” (0 marks). Scores for each subsection were summed, and the total scores were classified into 3 categories: poor practices (less than 60%), fair practices (60% to <75%), and good practices (75% or more).

#### Self-Care Skills Checklist

The researchers used the self-care skills checklist in phase II to evaluate participants’ practical self-care skills through direct observation. This assessment focused on 3 key tasks: preparing and injecting insulin (comprising 9 and 7 steps, respectively) and testing glucose levels in urine (9 steps). Conducting direct observations and assessments postintervention enhanced objectivity, providing a more reliable evaluation compared to patients’ subjective self-ratings. The checklist used a 3-point scoring system for each step: 3 points for correctly performed steps, 2 points for incorrectly performed steps, and 1 point for steps not performed. Subtotal scores for each skill were calculated, and participants’ performance was categorized as either satisfactory (≥60%) or unsatisfactory (<60%) based on the total score for each individual skill and the overall score.

#### Language of Data Collection Tools

Questionnaires in this study were used to accommodate the linguistic and practical needs of the participants and researchers. All questionnaires, except the self-care skills checklist, were in Arabic to ensure clear and effective communication with the study participants, who are native Arabic speakers. Delivering the questionnaire in their native language facilitated accurate comprehension of the questions and reliable responses, minimizing the risk of misinterpretation. The self-care skills checklist was in English, as it was designed for and completed by the researchers, all of whom possess a high level of proficiency in English. Using English for the researcher-administered questionnaire allowed for precision in recording and interpreting data while maintaining consistency with standard scientific and academic conventions. This dual-language approach ensured that both participants and researchers could engage effectively with the study materials, optimizing the validity and reliability of the data collected.

#### Piloting and Validation

The developed tools were validated by a panel of experts from the Faculty of Nursing at Kafr El-Shiekh University. The panel consisted of 5 experts: 2 professors of medical-surgical nursing, 1 professor of medicine from the Faculty of Medicine, 1 assistant professor, and 1 lecturer of medical-surgical nursing from the Faculty of Nursing. The tool underwent both face and content validity assessments. The content validity focused on evaluating the clarity, appropriateness, applicability, wording, and comprehensiveness of the tool. To assess the internal consistency of the tool, the Cronbach α test was used. The results showed a Cronbach α of 0.78 for the knowledge assessment questionnaire, 0.8 for the reported self-care practice scale, and 0.88 for the diabetic self-care practice checklist. The same group of experts also validated the scientific content of the educational program.

After incorporating the experts’ recommendations, the questionnaire was pilot tested. The pilot study was conducted over 3 weeks and included 10% (40/400) of the sample size (40 patients with DM in phase I and 10 patients in phase II). The purpose of the pilot study was to evaluate the clarity, applicability, and comprehensiveness of the tools and to assess the feasibility of the study process. Based on the findings from the pilot study, necessary modifications were made, such as the omission or addition of certain questions, to enhance the content, improve simplicity and clarity, and ensure the tools were concise and focused. The patients who participated in the pilot study were excluded from the main study sample.

#### Data Collection Process

**Figure 1.**
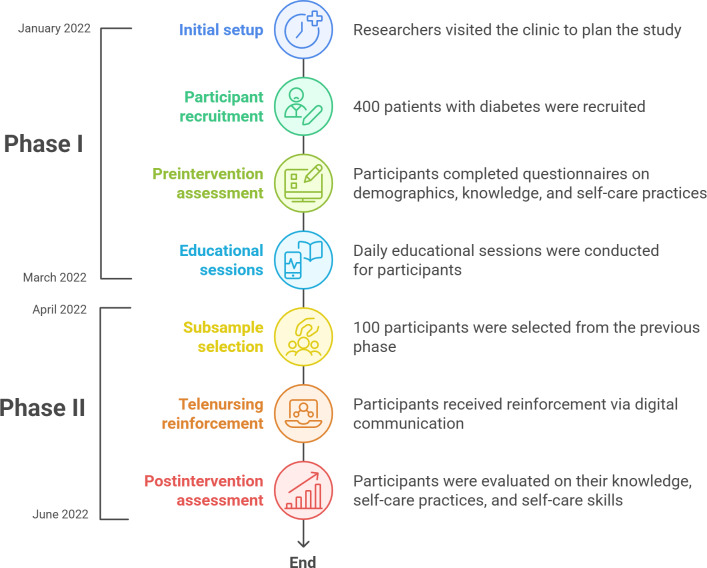
Data collection process.

Data collection spanned approximately 6 months, from January 2022 to the end of June 2022, and consisted of 2 phases as shown in Figure 1.

##### Phase I

The researchers first visited the diabetic clinic to discuss the research objectives and methods with nursing leaders. During this visit, they coordinated meetings with potential participants and identified private spaces for conducting interviews and delivering the initial intervention sessions. A sample of 400 patients with DM, meeting the previously described inclusion criteria, was selected to explore their knowledge of DM and self-care practices. Potential participants were informed about the study’s purpose and invited to participate if they were interested in receiving the intervention. Data collection took place for those who agreed to participate, with each interview lasting between 15 and 30 minutes (approximately 10‐14 participants were interviewed per day, one-on-one).

Participants were grouped according to their outpatient appointments, and the educational intervention sessions were conducted in groups ranging from 10 to 15 members. Sessions were held daily, excluding Fridays, with each session lasting 30‐40 minutes. Each session began with a welcome and icebreaker, followed by an explanation of the session’s objectives and topics, and concluded with a recap and time for participants to ask questions. The sessions ended with an open discussion, allowing participants to address any clarifications, and handouts related to the content were distributed for participants to read at their convenience.

##### Phase II

As mentioned earlier, a purposive sample of a hundred patients from phase I was selected to undergo phase II. Participants underwent telenursing reinforcement of the educational intervention content from phase I via calls, SMS text messages, WhatsApp messages, videos, and voice notes. This was undertaken over 4 weeks (details of this intervention are provided in the following section). One month after the telenursing reinforcement, a posttest assessment was carried out at the outpatient clinic in Kafr Elshiekh Hospital. During this assessment, patients were interviewed to evaluate their knowledge, reported self-care practices, and self-care skills.

### Intervention

#### Intervention Design

The intervention in this study was developed by the researchers, all of whom were diabetes nursing specialists, to enable patients with DM to enhance their self-care practices in response to the needs of patients. The intervention included educational sessions and telenursing follow-ups as described below.

#### Educational Sessions

The researchers delivered 6 educational sessions to the 400 participants in phase I at Kafr El Sheikh University Hospital’s outpatient diabetes clinic. The educational sessions were held daily, excluding Fridays, with each session lasting 30‐40 minutes. The educational sessions involved 3 theoretical and 3 practical sessions on diabetes. Specifically, the theoretical sessions covered essential topics such as basic knowledge of DM and its management (including medical treatment, physical exercise, dietary management, foot care, follow-up, and lifestyle habits that exacerbate the disease). The practical sessions focused on promoting healthy lifestyles (eg, dietary practices and physical exercises) and self-care practices (eg, insulin preparation and injection, glucose testing in urine, blood glucose monitoring, medication schedules, prevention of complications, and foot care practices).

To reinforce the learning experience, the educational content was compiled into a booklet distributed to participants after the sessions, serving as a reference for the information provided. A variety of pedagogical methods were used during the sessions, including practical demonstrations, abstract lectures, group discussions, and role-playing activities. These diverse teaching styles were designed to accommodate different learning preferences and build participants’ confidence and adherence to the intervention. Additionally, visual aids such as images, physical models, and PowerPoint presentations were used to enhance understanding and engagement throughout the sessions.

#### Telenursing Follow-Up

During phase II, 100 participants were purposively selected for a 4-week telenursing follow-up. This intervention aimed to reinforce the educational content provided in phase I and support patients in adopting effective self-care practices. The follow-up schedule included daily 10‐15-minute calls in the first week, twice-weekly calls in the second week, and weekly calls in the final 2 weeks. These personalized interactions focused on revisiting the educational material and addressing any questions or challenges faced by the participants.

During the follow-up period, participants received daily health education through various channels, including SMS text messages, WhatsApp messages, voice notes, and videos, all of which reiterated the information provided in the educational booklet. To further encourage adherence to self-care practices, daily reminders and audio recordings were sent to prompt actions such as blood glucose self-assessment, medication compliance, foot care, physical activity, and following the recommended diet plan. The content of the telenursing follow-up was meticulously developed by the researchers, drawing on insights from phase I, evidence-based diabetes self-care guidelines, and input from diabetes nursing specialists and clinical researchers. This ensured the content was accurate, culturally relevant, and aligned with the specific needs of the participants.

### Statistical Analysis

Data collection, coding, and analysis were undertaken using SPSS (version 20, IBM Inc). Mean and SD values were used to report continuous data (with independent sample *t* testing to compare group differences) and frequency and percentage values for categorical data (with chi-square and Fisher exact probability tests to determine intervariable relationships). Pretest-posttest differences were determined using paired sample *t* tests and the McNemar test, indicating binary categorical variables’ changes following the intervention. The robustness of the contingency table analyses was assured using Monte Carlo simulations. The application of these methods of statistical analysis affirmed cross-comparison results’ reliability and mitigated risks of erroneously rejecting null hypotheses, as presented in the following section. After adjustments, a *P* value of ≤.05 was assumed to indicate statistical significance.

### Ethical Considerations

Kafr El Sheikh University granted ethical approval for this study (KFIRB200-9). Studied patients gave verbal consent to taking part after full disclosure of the nature and scope of the research and their rights, including their ability to decline to take part or to subsequently withdraw without any consequences for their health care services or statutory rights. They were assured of their right to confidentiality, and that all data are reported anonymously in this study, with coding. All participants were informed that the data related to their participation would only be used for the current research purpose as per ethical guidelines for participant protection.

## Results

### Overall Findings

As described in the following subsections, significant shortcomings were discovered in participants’ knowledge and skills at baseline, especially for female patients. The results after the intervention revealed significant enhancements in self-care practice and knowledge scores (*P*<.001). All of the patients were able to ascend from “unsatisfactory” to “satisfactory” scores in relation to skills for DM self-care, underscoring the efficacy of the intervention in enabling patients to achieve improved self-management of DM.

### Sociodemographic Characteristics

The total studied sample comprised 400 people with DM, who had a mean age of 49.7 (SD 11.5) years. As shown in Table 1, the largest cohort (143/400, 35.7%) was aged 55‐65 years, while over a fifth (86/400, 21.5%) each were aged 35‐44 and 45‐54 years. The vast majority of patients resided in family residences (399/400, 99.7%) and were married (325/400, 81.3%). A large minority (149/400, 37.2%) reported being illiterate, while almost a third (130/400, 32.5%) cited secondary school as their highest educational level and a negligible proportion (12/400, 3%) reported being university-educated. Females were significantly more likely to be illiterate (108/200, 54%) than males (41/200, 20.5%*; P*<.001). Furthermore, a negligible proportion (1/200, 0.5%) of male participants were unemployed, while none of the female participants were employed.

**Table 1.** Participants’ sociodemographic characteristics.

Variables	Male (n=200)	Female (n=200)	Total (N=400)	*χ* ^2^ ^a^	*P* value
Age (years), n (%)				4.6	<.001
29‐34	42 (21)	16 (8)	58 (14.5)		
35‐44	41 (20.5)	45 (22.5)	86 (21.5)		
45‐54	40 (20)	46 (23)	86 (21.5)		
55‐64	69 (34.5)	74 (37)	143 (35.7)		
65‐72	8 (4)	19 (9.5)	27 (6.8)		
Mean (SD)	47.1 (11.4)	52.3 (11.1)	49.7 (11.5)		
Marital status, n (%)				9.2	.1
Single	8 (4)	3 (1.5)	11 (2.7)		
Married	170 (85)	155 (77.5)	325 (81.3)		
Widowed	22 (11)	42 (21)	64 (16)		
Living alone?, n (%)				-	.317^b^
Yes	0 (0)	1 (0.5)	1 (0.3)		
No	200 (100)	199 (99.5)	399 (99.7)		
Education, n (%)				59.2	<.001
Illiterate	41 (20.5)	108 (54)	149 (37.2)		
Literate	41 (20.5)	39 (19.5)	80 (20)		
Preparatory	21 (10.5)	8 (4)	29 (7.3)		
Secondary	87 (43.5)	43 (21.5)	130 (32.5)		
University	10 (5)	2 (1)	12 (3)		
Work status, n (%)				-	<.001^c^
Working	199 (99.5)	0 (0)	199 (49.7)		
Not working	1 (0.5)	200 (100)	201 (50.3)		

a Two-tailed.

b *P* value for Living alone? is based on Fisher exact test.

c *P* value for Work status is based on Monte Carlo exact test.

### Clinical Characteristics

As Table 2 shows, one-third (135/400, 33.8%) of patients in this study received their DM diagnosis over 10 years previously, and the majority (231/400, 57.7%) had the comorbidity of cardiac disease. Concerning the latter condition, males (82/200, 41%) were significantly less likely to have it than females (149/200, 74.5%*; P*<.001). Almost half (176/400, 44%) of patients just received insulin treatment, while almost a quarter (93/400, 23.3%) additionally received oral hypoglycemic medications. The vast majority of patients exhibited elevated blood glucose (365/400, 91.3%), and most were overweight (146/400, 36.5%) or obese (147/400, 36.8%); females were disproportionately more prone to obesity (135/200, 67.5%) than their male counterparts (12/200, 6%).

**Table 2.** Participants’ medical symptoms.

Medical data	Male (n=200), n (%)	Female (n=200), n (%)	Total (N=400), n (%)	*χ* ^2^ ^a^	*P* value
**Disease onset (years)**				22.8	<.001
<1	28 (14)	42 (21)	70 (17.5)		
1‐5	34 (17)	49 (24.5)	83 (20.7)		
5‐10	48 (24)	64 (32)	112 (28)		
10+	90 (45)	45 (22.5)	135 (33.8)		
**Other chronic diseases**					
None	117 (58.5)	51 (25.5)	168 (42)	5.9	<.001
Cardiac disease	82 (41)	149 (74.5)	231 (57.7)	4.2	<.001
Hypertension	19 (9.5)	6 (3)	25 (6.2)	1.7	.541
Renal disease	0 (0)	2 (1)	2 (0.5)	0.51	.814
Rheumatic disease	2 (1)	1 (0.5)	3 (0.7)	0.5	.885
Liver disease	5 (2.5)	4 (2)	9 (2.2)	0.1	.924
**Type of diabetes treatment regimen**				28.2	<.001
Oral hypoglycemic drugs	65 (32.5)	66 (33)	131 (32.7)		
Insulin	107 (53.5)	69 (35.5)	176 (44)		
Both	28 (14)	65 (32.5)	93 (23.3)		
**Commitment to follow-up schedule**				
Always	200 (100)	200 (100)	400 (100)	N/A^b^	N/A
**Fasting blood glucose**				0.83	.662
Below normal	1 (0.5)	1 (0.5)	2 (0.5)		
Normal	14 (7)	19 (9.5)	33 (8.2)		
Above normal	185 (92.5)	180 (90)	365 (91.3)		
**BMI**				94.2	<.001
Normal	79 (39.5)	28 (14)	107 (26.7)		
Overweight	109 (54.5)	37 (18.5)	146 (36.5)		
Obese	12 (6)	135 (67.5)	147 (36.8)		

a Two-tailed.

b N/A: not applicable.

### Baseline Knowledge Scores

In terms of knowledge, the majority (255/400, 63.7%) exhibited poor knowledge at baseline, albeit this was significantly less pronounced among males (102/200, 51%) than females (153/200, 76.5%; *P*<.001), as shown in Table 3. About half (98/200, 49%) of male participants had “fair” knowledge, while less than a quarter (47/200, 23.5%) of females did. Consequently, the outcomes underscore major differences in baseline knowledge among males and females, especially concerning comprehension of appropriate DM management practices, as affirmed by results on actual practices (discussed below), indicating the necessity of specific educational interventions targeted to females.

**Table 3.** Baseline diabetes mellitus knowledge.

	Male (n=200), n (%)	Female (n=200), n (%)	Total (N=400), n (%)	*χ* ^2^	*P* value
Knowledge level				28.1	<.001
Poor (<60%)	102 (51)	153 (76.5)	255 (63.7)		
Fair (≥60%)	98 (49)	47 (23.5)	145 (36.3)		

### Baseline Self-Care Practices

At the beginning of the intervention, most patients (248/400, 62%) exhibited inadequate baseline self-care practices, albeit this was significantly lower among males (96/200, 48%; *P*<.001) than females (152/200, 76%), as shown in Table 4. “Good” practices for self-care were only reported among 24% (48/200) of males and 8% (16/200) of females. The lowest adherence was noted for blood glucose monitoring (316/400, 79%), physical exercise (296/400, 74%), and the prevention and management of acute complications (268/400, 67%). Critical shortfalls in self-care behaviors were thus observed, especially with regard to females, which indicates that more targeted interventions are needed to enhance essential self-care among female service users (in addition to the general need for improved self-care among DM patients in general).

**Table 4.** Pretest self-care practice scores.

	Male (n=200), n (%)	Female (n=200), n (%)	Total (N=400), n (%)	*χ* ^2^	*P* value
Total practice score				35.2	<.001
Good (≥75%)	48 (24)	16 (8)	64 (16)		
Fair (60‐74%)	56 (28)	32 (16)	88 (22)		
Poor (<60%)	96 (48)	152 (76)	248 (62)		

### Intervention Impacts on DM Knowledge

Table 5 demonstrates that the intervention achieved significant enhancements of patients’ DM management knowledge for all studied domains (*P*<.001). The biggest improvements were seen concerning physical exercise knowledge, which saw a mean increase of 6.6 points, and tangible improvements were seen in knowledge of dietary choices and regimens of treatment.

**Table 5.** Mean of diabetes mellitus knowledge scores before and after the intervention (n=100).

Knowledge domains	Score, mean (SD)		Mean change^a^	*t* test^b^	*P* value
	Preintervention	Postintervention			
Basic knowledge about DM^c^	12.1 (3.7)	40.3 (7.4)	28.2	31.8	<.001
Treatment regimen	6.2 (1.9)	10.5 (1.5)	4.3	9.5	<.001
Physical exercise	1.2 (0.5)	8.2 (1.9)	7	37.4	<.001
Importance of follow-up visits	5 (0)	5.8 (0.4)	0.8	21.9	<.001
Dietary knowledge	3 (1.3)	6.5 (1.2)	3.5	22.9	<.001
Foot care knowledge	1.4 (0.6)	3.8 (0.6)	2.4	25.6	<.001
Knowledge of bad habits increasing DM severity	2.1 (0.5)	3.8 (0.5)	1.7	23.5	<.001
Total knowledge score	29.6 (3.9)	71.8 (13)	42.2	30.7	<.001

a Mean change = Posttest score – Pretest score.

b Two-tailed paired sample *t* test.

c DM: diabetes mellitus.

### Intervention Impacts on Self-Care Practices

As shown in Table 6, the applied intervention achieved statistically significant enhancements of practices for self-care for all studied domains (*P*<.001). Mean increases of 8.15 points each were attained for the practices of “foot care” and “blood glucose monitoring,” with a more modest increase in exercise practices of 3.45 points. These outcomes indicate that the intervention successfully improved participants’ self-care behaviors for improved DM management.

**Table 6.** Mean of self-care practice scores before and after the intervention (n=100).

Practice domains	Score, mean (SD)		Mean change^a^	*t* test^b^	*P* value
	Preintervention	Postintervention			
Nutritional practices	8.4 (2.2)	21.2 (2.3)	12.8	40.4	<.001
Treatment regimen adherence	2.3 (0.6)	7 (0)	4.7	55.4	<.001
Monitoring of blood glucose level	1.6 (0.9)	10.3 (2.5)	8.7	36.9	<.001
Physical activities	3.8 (1.7)	14.2 (1.1)	10.4	55.1	<.001
Practices to avoid complications	5.2 (2)	10.2 (1.2)	5	21.9	<.001
Foot care practices	1.6 (0.9)	10.3 (2.5)	8.7	36.9	<.001
Total practice score	20.8 (4.5)	59.9 (7)	39.1	53.7	<.001

a Mean change = (Posttest score – Pretest score)/Pretest score.

b Two-tailed paired sample *t* test.

### Intervention Impacts on Self-Care Skills

Significant improvements were seen following the intervention in patients’ self-care skills, as shown in Table 7. Every participant went to “satisfactory” postintervention from “unsatisfactory” at baseline (400/400, 100%*; P*<.001), as reflected in the baseline scores for insulin preparation (mean 12.3, SD 2.1), self-injection (mean 11.8, SD 2.5), and glucose testing (mean 10.5, SD 1.9) increasing to 25.4 (SD 3), 24.8 (SD 2.8), and 23.5 (SD 2.4), respectively. These results underscore the effectiveness of the intervention to empower patients with prerequisite DM management skills, demonstrating the efficacy of practical instruction and reminders and reinforcement via telenursing, with the possibility of scalability for different and varied populations.

**Table 7.** Level of self-care skills before and after the intervention.

Self-care skills	Preintervention	Postintervention	*χ* ^*2*^ ^a^	*P* value
**Insulin preparation (n=58), n (%)**			47	<.001
Unsatisfactory	58 (100)	0 (0)		
Satisfactory	0 (0)	58 (100)		
**Insulin injection (n=58), n (%)**			47	<.001
Unsatisfactory	58 (100)	0 (0)		
Satisfactory	0 (0)	58 (100)		
**Urine glucose testing (n=100), n (%)**			51.9	<.001
Unsatisfactory	100 (100)	0 (0)		
Satisfactory	0 (0)	100 (100)		

a McNemar test for related groups.

## Discussion

### Main Outcomes

#### Summary of Key Findings

This study on patient education and telenursing impacts concerning self-care practices among DM patients produced statistically significant outcomes, encompassing quantifiable improvements in skills, knowledge, and practices. Consequently, the intervention was effective in improving DM self-management and mitigating risks, as described below.

#### Improved DM Knowledge

The intervention resulted in patients with DM attaining significantly improved DM knowledge, especially concerning physical exercise, nutrition, and compliance with treatment. A more in-depth understanding of physical exercise was reflected in the postintervention increase in mean knowledge about physical exercise (and its impact on blood glucose) by 6.6 points [13]. This was striking, as education for patients with DM often lacks sufficient attention to physical exercise, despite its fundamental place in managing blood glucose and avoiding DM complications [13]. Improved knowledge scores concerning regimens and nutrition were also significant, and these outcomes are essential for the strategy of managing diabetes.

The intervention analyzed in this research effectively addressed existent educational needs among DM patients, offering them accurate and clear information they could apply, via easy-to-use formats (eg, SMS text messages, telephone calls, and WhatsApp). The ease of access enabled patients to effectively manage their conditions, which was particularly useful for the subset recruited for phase II, due to their particularly poor knowledge and self-care determined in the preliminary assessment. Although individual needs of specific patients were not targeted by the studied intervention, it was directed to commonly identified barriers and needs among patients requiring such services, offering scope for genuine enhancements in patients’ outcomes and self-care behaviors.

#### Improved Self-Care Practices

Self-care practices significantly increased participants’ scores for practices following the intervention, including monitoring blood glucose, undertaking appropriate foot care, and physical exercise. For monitoring blood glucose and foot care, participants achieved a mean improvement of 8.15 points each, highlighting the efficacy of the intervention in terms of encouraging positive practices to avoid long-term complications and deteriorating health conditions, including serious ones commonly affecting patients with DM due to a dearth of appropriate self-care (eg, neuropathy and foot ulcers) [14]. Physical exercise-related self-care practices also yielded an improvement of 3.45 points, showing more likelihood of undertaking exercise after the intervention. This addresses a core aspect of the management of diabetes, enhancing sensitivity to insulin and lowering the risk of cardiovascular damage [15].

#### Improved Self-Care Skills

The effects of the intervention on participants’ self-care skills were substantial; all 100% (400/400) had “unsatisfactory” skills preintervention, and 100% (400/400) had “satisfactory” skills after it, in terms of preparing and injecting insulin and testing glucose in urine. This demonstrates the potentially remarkable effectiveness of patient education and telenursing follow-ups to enable patients with DM or other serious conditions to more proactively improve and maintain positive skills and behaviors, thereby improving their health outcomes (and substantially reducing costs for health systems).

#### Relation to Existing Literature

The outcomes of this study affirm those of the broader literature on positive telenursing impacts on the management of chronic diseases, such as DM [6,16]. Previous studies have extensively demonstrated particular impacts of telenursing in terms of enhanced engagement and medication adherence among patients, which ultimately contribute to improved prognosis [17,18]. This research contributes to the literature by presenting how a holistic telenursing intervention combining educational with skills-based content delivered via modern telecommunications (eg, WhatsApp messages) can facilitate major breakthroughs for patients in terms of increased self-care practices and DM management knowledge. This notably goes beyond most DM-related research, which tends to prioritize fundamental biomedical indicators of telenursing effectiveness (eg, HbA_1c_ and BMI), without commensurate attention to the holistic dimensions of DM care and self-management for patients (eg, exercise) [8,19].

### Implications for Practice and Research

The intervention used in this research achieved notable benefits for patients, offering broader potential impacts for health practice and studies. For practitioners, the outcomes of this study affirm the effectiveness of patient education and telenursing follow-ups to improve diabetes care services, and personalized support and education delivered remotely via modern technologies, which are increasingly ubiquitous, can enlarge patient access to education and improve medication and healthy behavior adherence. Such impacts reduce demand for conventional clinical resources and avoid the escalation of negative DM-related conditions, thereby improving quality of care (ie, patient health and satisfaction) while achieving maximum resource deployment efficiency for health systems, which is essential for contexts with limited resources (eg, in low-income countries or remote geographical areas).

This research suggests that the effectiveness of patient education and telenursing follow-ups can be enhanced by adopting a patient-centered approach that addresses specific gaps in skills and knowledge among particular patient groups or individual patients. A personal paradigm considering each patient’s particular requirements, as applied in this study, can enable patient education and telenursing follow-ups to offer its full benefits, reducing net costs on conventional health care resources, especially for chronic and serious conditions requiring improved self-management by patients, such as DM. In terms of implications for research, this study leaves open the requirement to investigate longitudinal effects of patient education and telenursing follow-ups to see if the advantages for self-care practices and DM knowledge recorded after a few weeks in this research can be sustained over time, and the extent to which they affect health indicators over the longer term (eg, reduced rates of DM complications and improved glycemic control). Furthermore, the cost efficiency of patient education and telenursing follow-ups in various potential applications can be compared to enable policy development to optimally deploy such initiatives for the maximum benefits. Finally, future studies should consider conducting 2-arm RCTs to compare patient education and telenursing follow-ups with standard care. Research involving diverse populations would also help determine the broader applicability of this approach. Additionally, integrating mobile health applications with automated reminders could enhance communication and patient adherence, potentially improving self-care related outcomes.

### Limitations

The foremost limitation of this study pertains to its reliance on patients’ own self-perceived and rated performance for some of the tools used, which is obviously subject to various forms of bias (including social desirability bias concerning self-care practices when reporting data in health care contexts). Furthermore, it was not possible to objectively measure patient indicators outside of clinical settings, including their exercise habits, nutrition, and blood glucose levels; such data would have offered improved, robust proof concerning the positive impacts of the intervention.

The used design, with a single group and pretest-posttest format, precludes the use of a control group, which consequently reduces the confidence with which observed changes can be solely attributed to the intervention. It should also be remembered that many patients in real clinical contexts lack access to the internet, smartphones, etc, due to socioeconomic and geographical barriers and digital literacy, which can affect the applicability of this and other interventions, undermining the equity of telenursing care. The single setting from which participants were recruited is also an issue that reduces generalizability. It is advised that researchers use objective methods of measuring patients’ clinically relevant data in studies of their self-care behaviors, use control groups, and recruit participants from multiple contexts in order to generate more generalizable feedback about patient needs and the efficacy of interventions. Researchers should also always consider accessibility issues, including with regard to the use of digital technologies to deliver care.

### Conclusions

The results affirm that an appropriately designed educational telenursing intervention can achieve significantly improved patient knowledge, self-care practices, and skills among patients with DM. Delivered via numerous modern methods of telecommunication, the intervention was successful in targeting essential issues in DM management to prevent complications, including monitoring blood glucose, physical exercise, and appropriate care for the foot. These results buttress calls for telenursing inclusion in conventional care for patients with DM, especially in contexts where conventional resources are not optimally accessible for all patients. This study’s outcomes highlight the potential of patient education and telenursing follow-ups as an effective and scalable intervention to enable improved self-care practices, skills, and knowledge for patients with DM. The statistically significant enhancements demonstrated by this research support the use of patient education and telenursing follow-ups to help address the expanding costs of diabetes care, especially in contexts with limited resources. Nevertheless, more studies are required to ascertain whether the outcomes of this study are similar across different service user populations and to assay the long-term clinical and economic sustainability of education and telenursing solutions for such care.

## Abbreviations

DM: diabetes mellitus
HbA_1c_: glycated hemoglobin A_1c_
RCT: randomized controlled trial
